# Prenatal ultrasonographic diagnosis of cleft lip with or without cleft palate; pitfalls and considerations

**DOI:** 10.1186/s40902-015-0019-z

**Published:** 2015-08-14

**Authors:** Dong Wook Kim, Seung-Won Chung, Hwi-Dong Jung, Young-Soo Jung

**Affiliations:** 1 Department of Oral and Maxillofacial Surgery, Oral Science Research Institute, Yonsei University College of Dentistry, 50-1 Yonsei-ro, Seodaemun-gu, Seoul, 120-752 Republic of Korea; 2 Graduate School, Yonsei University College of Dentistry, 50-1 Yonsei-ro, Seodaemun-gu, Seoul, 120-752 Republic of Korea; 3 Department of Dentistry, CHA Bundang Medical Center, CHA University, 59 Yatap-ro, Bundang-gu, Seongnam-si, 463-712 Gyeonggi-do Republic of Korea

**Keywords:** Cleft lip, Cleft palate, Prenatal ultrasonography

## Abstract

Ultrasonographic examination is widely used for screening of abnormal findings on prenatal screening. Cleft lip with or without cleft palate of the fetus can also be screened by using ultrasonography. Presence of abnormal findings of the fetal lip or palate can be detected by the imaging professionals. However, such findings may not be familiar to oral and maxillofacial surgeons.

Oral and maxillofacial surgeons can use ultrasonographic imaging of fetal cleft lip with or without cleft palate to provide information regarding treatment protocols and outcomes to the parent. Therefore, surgeons should also be able to identify the abnormal details from the images, in order to setup proper treatment planning after the birth of the fetus.

We report two cases of cleft lip with or without cleft palate that the official readings of prenatal ultrasonography were inconsistent with the actual facial structure identified after birth. Also, critical and practical points in fetal ultrasonographic diagnosis are to be discussed.

## Background

The incidence of the various types of cleft lip (CL) with or without cleft palate (± CP) is 1 per 700~1000 live births worldwide [1, 2]. Cleft lip with or without cleft palate (CL ± CP) is the most common fetal craniofacial malformation that is screened during prenatal ultrasonographic examination [3].

An accurate prenatal diagnosis of the lip and palate anomaly is critical for establishing adequate long-term treatment planning, prediction of prognosis, and proper counseling with the parent [4]. Studies reporting the accuracy of 2D ultrasonography in detecting CL ± CP in low-risk populations demonstrate a wide variety in diagnostic accuracy [5]. The sensitivity of routine transabdominal ultrasonic scan at 20-weeks’ gestation varies from 16 % to 93 %, indicating a considerable proportion of misdiagnosis [5–8].

Meanwhile, health professionals are expected to have accurate and clear answers on possible questions from the parents following ultrasonographic examination [4, 5]. Thus when the surgeons are to make treatment planning and counsel the parents, they should be aware of the potential pitfalls of the ultrasonography [4], as there can be possible inconsistencies between reading from the imaging professionals and the actual fetal facial structural anomalies.

Here we report two cases of CL with or without CP that the official readings of prenatal sonography and the actual facial structure identified after birth were somewhat inconsistent. After the case review, we would like to discuss the points to be considered in fetal ultrasonographic diagnosis.

## Case presentation

### Case 1

**Fig. 1:**
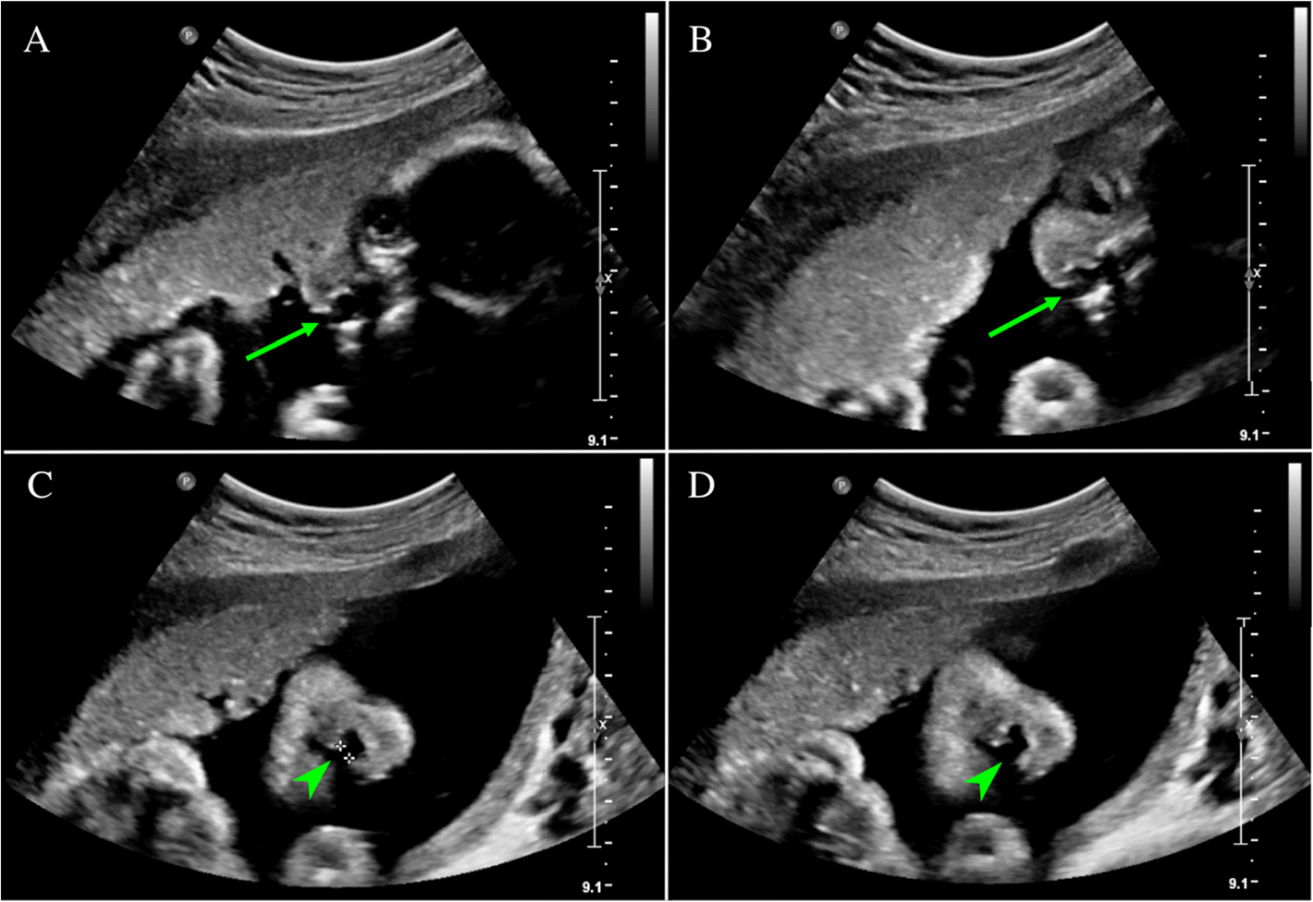
Transabdominal ultrasonography of Case 1, 26 weeks’ gestation. **a**, **b**: Arrow indicates the cleft palate site. **c**, **d**: Arrowhead indicates the cleft lip site

A 40-year-old healthy multipara was referred from local obstetrics and gynecology (OBGYN) clinic to the department of OBGYN in Severance hospital for further evaluation and management of congenital heart defect and unilateral CL + CP detected on ultrasonography at 26 weeks’ gestation. The patient was referred to our department for prenatal counseling and treatment planning for the unilateral CL + CP (Fig. 1).

**Fig. 2:**
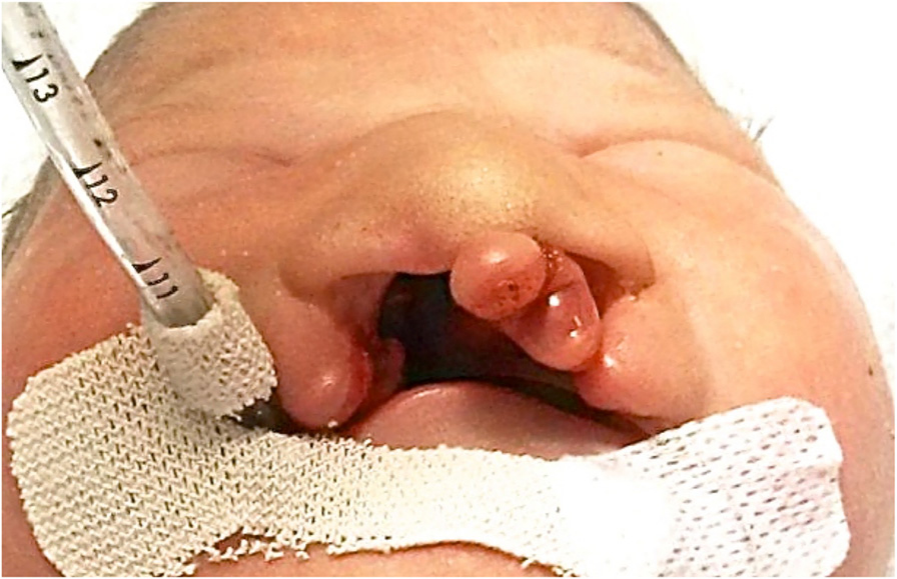
Case 1, after birth

After birth, the newborn was confirmed to have bilateral complete CL + CP, which was much more severe than that had been predicted (Fig. 2). Prenatally diagnosed congenital heart defect (double outlet right ventricle with ventricular septal defect) was also confirmed.

### Case 2

**Fig. 3:**
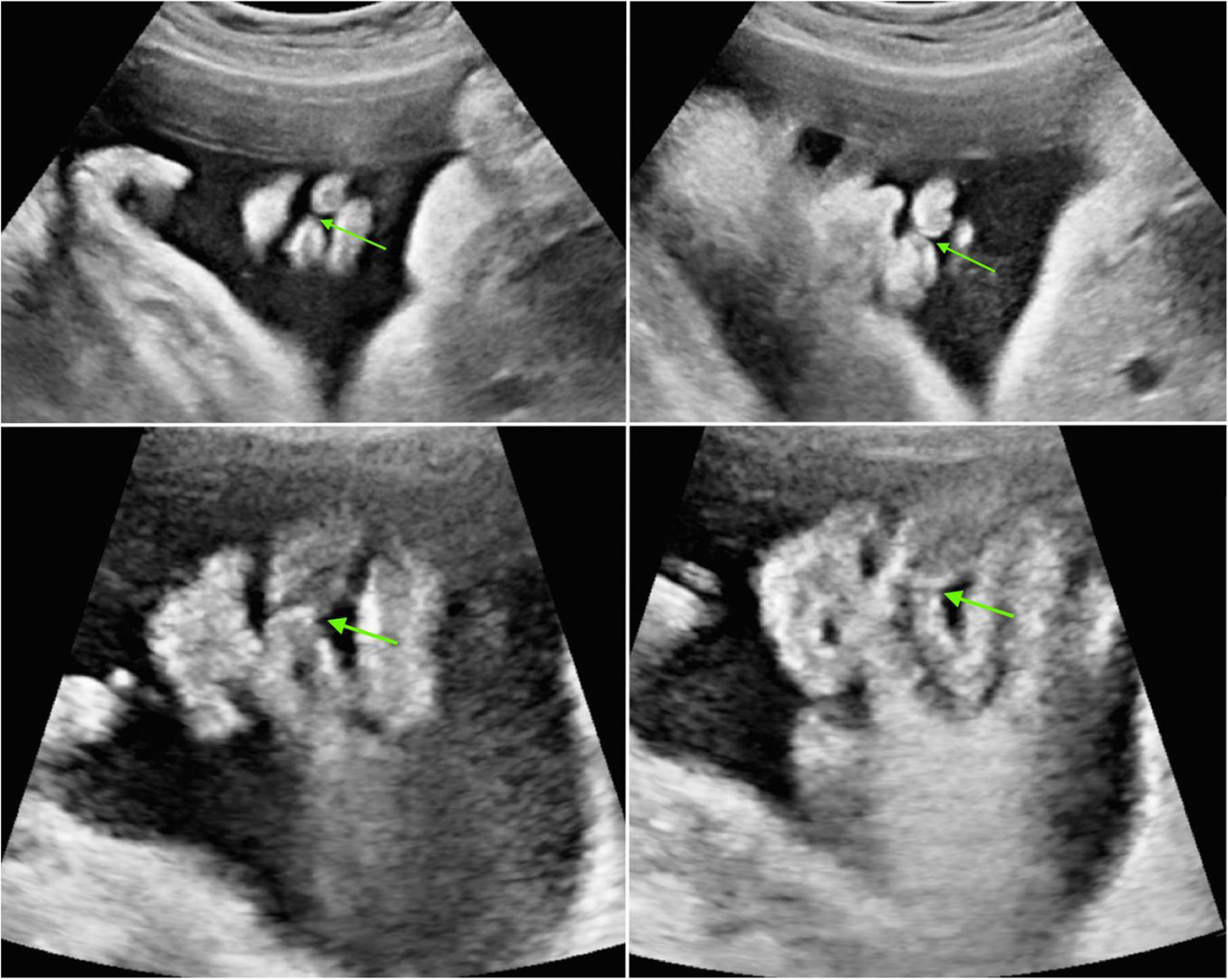
Transabdominal ultrasonography of Case 2, 35 weeks’ gestation. Arrows indicate cleft lip site

**Fig. 4:**
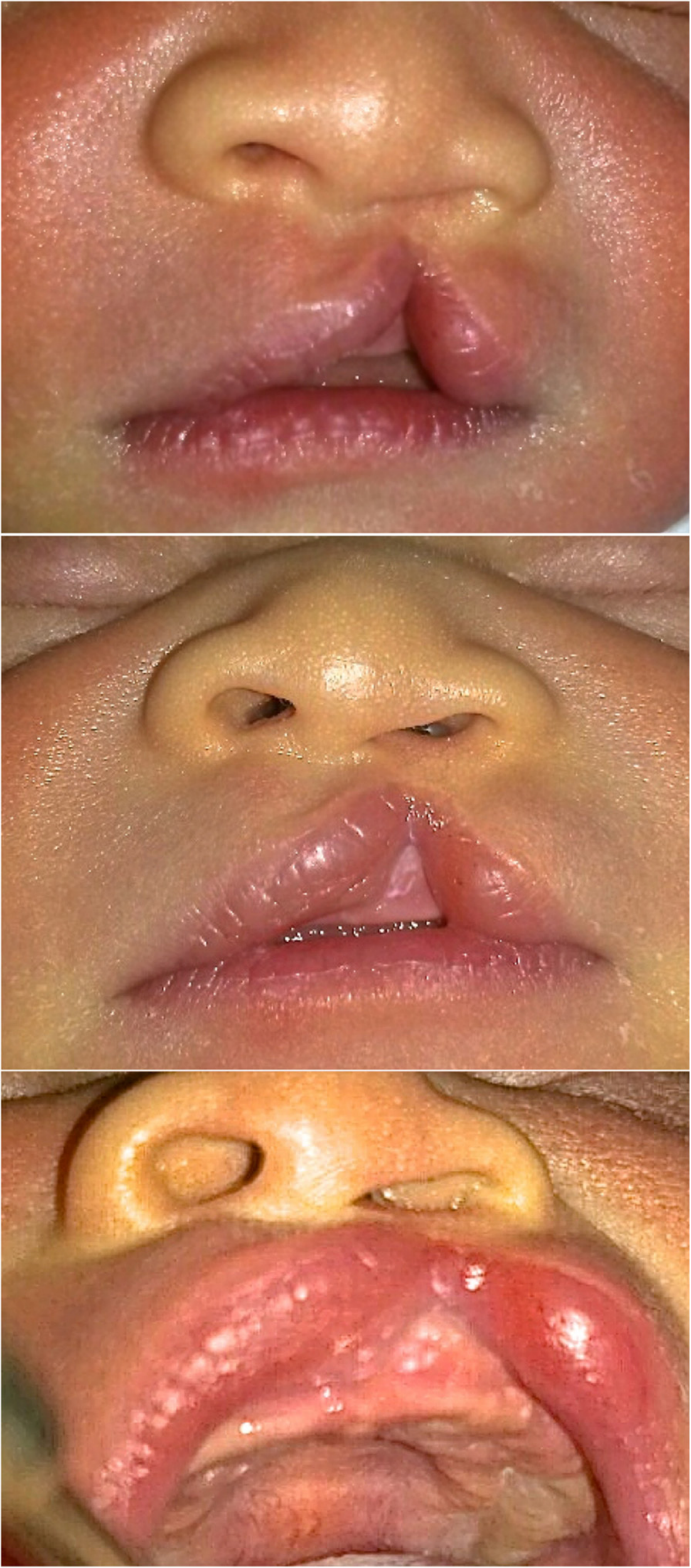
Case 2, after birth

A 29-year-old nullipara was under routine follow-up in the department of OBGYN in Severance hospital until 20 weeks’ gestation. Until then, there were no known problems regarding the mother and the fetus, and the there were no detected anomalies in ultrasonography. From then, the mother was lost to follow-up. On 35 weeks’ gestation, she revisited department of OBGYN in Severance hospital, referred from local OBGYN clinic due to cleft lip of the fetus detected on ultrasonography. Ultrasonographic examination in the department of OBGYN in our hospital revealed unilateral cleft lip (Fig. 3). After birth, the newborn was confirmed to have isolated incomplete CL (Fig. 4).

## Conclusion

The Case 1 shows an example of actual anomaly confirmed after birth being severe than it was predicted based on the ultrasonography. In the case 2, cleft lip was detected relatively late in gestational period. We would like to discuss the factors affecting prenatal ultrasonographic diagnosis, and special considerations of cleft lip with or without palate regarding such cases.

### Accuracy of the prenatal ultrasonography

The accuracy of sonography for prenatal diagnosis of CL ± CP is highly variable and dependent on the experience of the sonographer, maternal body habitus, gestational age, fetal position, and the amount of amniotic fluid and the type of clef [4, 9, 10].

Cash et al. reported that when the overall detection rate for facial clefts was 65 %, the detection rate for CL with CP was 93 %, isolated CL was 67 %, and isolated CP was 22 % [8]. Isolated cleft palate is reported to be rarely identified prenatally [6, 9]. Therefore, surgeons should to be aware that a negative ultrasound result does not necessarily mean that unborn child is without orofacial cleft. Transabdominal 2D ultrasonographic screening for orofacial clefts in a low-risk population has a relatively low detection rate and low false-positive diagnosis [5].

### Types of orofacial cleft

It is important to differentiate the various types of orofacial clefts, because each type of orofacial cleft has different prognosis [5, 11]. For instance, when a CL is visualized on ultrasonography, it is difficult to determine whether the alveolus and secondary palate are involved [9]. When palate is involved, reconstruction technique, surgical implications would be more complicated, and the risk for chronic otitis media, hearing loss, abnormal speech, and midfacial retrusion would increase [9]. This can be applied to the case 1 above, as the prenatal diagnosis was unilateral CL and it turned out to be bilateral complete CL with CP. Meanwhile, CL without CP is associated with a relatively favorable prognosis [11].

Unilateral incomplete CL can be subtle and undetectable until the third trimester [5]. While complete orofacial clefts can be detected in ultrasonographic examination as early as 16 weeks’ gestation, unilateral incomplete CL is known to be detected after 27 weeks’ gestation, as in Case 2 [3, 9]. This minor labial clefting is not usually associated with other malformations and has relatively favorable prognosis [9, 11, 12]. At times, distinguishing between an incomplete and complete CL is difficult because there can be a thin band of tissue spanning the cleft even with a complete alveolar cleft [9, 13].

Even when a CL is visualized in ultrasonographic examination, it is difficult to determine whether the palate is also involved [9]. Approximately 90 % of fetuses with a complete cleft of the primary palate will also have a complete cleft of the secondary palate [9]. Conversely, 10 % of infants with complete unilateral or bilateral CL will have an intact secondary palate [9]. It is reported that reconstructed axial images obtained by 3D ultrasound of the fetal palate has high accuracy in identifying prenatal cleft palate when cleft lip is diagnosed at mid-trimester 2D ultrasound screening [14].

### Advanced imaging modalities – 3D ultrasonography and MRI

3D ultrasonography and prenatal MRI would improve the accuracy of prenatal diagnosis of orofacial clefts. 3D ultrasonography can provide more precise image of the defect and it has shown to enhance 2D examination significantly [5, 15–17]. While 3D ultrasonography can achieve a reliable diagnosis of fetal CL ± CP, this does not rule out cases of CP only [5].

Magnetic resonance imaging (MRI) has been useful for prenatal diagnosis of fetal deformities compared with US, adding valuable information or supplying higher diagnostic accuracy [12]. The use of MRI is increasing for evaluation of fetal abnormalities that are difficult to identify on ultrasonography alone [9]. Fetal MRI is less dependent than ultrasonography on optimal amniotic fluid volume, fetal position, and maternal body habitus [9]. Additionally, visualization of small structures on MRI is not limited by bone shadowing [9]. MRI enables visualization of the anterior six tooth buds (four of which arise from the premaxillary segment); and the continuous, smooth, echogenic, and horseshoe-shaped curve of the tooth-bearing alveolar ridge. This allows the diagnosis of alveolar cleft, and missing teeth buds, prenatally [9].

### Associated anomalies

In the prenatal population, fetus with CL + CP or cleft secondary palate usually has chromosomal abnormalities or other anomalies incompatible with survival [9]. Thus many fetuses with CL + CP die in utero or are spontaneously aborted and are never seen as newborns [3]. Therefore, the incidence of CL ± CP in the prenatal population is higher than that of postnatal population [9]. Thus prenatal ultrasonographic examination underscores the high incidence of spontaneous fetal loss that occurs whenever CL ± CP is associated with aneuploidy or other malformations [3].

Many syndromes have cleft as a part of their phenotypes and a cleft may be the only sign of a potential serious aneuploidy, as in above case 1 [18]. Severe additional anomalies can result in poor outcome, such as mortality [19]. Non-syndromic isolated CL ± CP has low mortality and morbidity rates and are primarily a functional and esthetic problem [5]. CL only is associated with a very small percentage of chromosomal anomalies, as in Case 2 [18]. Ultrasonographic examination can never rule out a chromosomal aberration. Therefore, patients should receive genetic counseling and should be offered karyotypic analysis of their fetus, if needed [4].

### Counseling the parents

Majority of the pregnant women and their partners are not expecting to identify a birth defect [4]. The initial shock caused by the discovery of a cleft is followed rapidly by fear, anger, sadness and guilt particularly if the baby is the first one [2, 4]. Prenatal diagnosis poses many challenges for professionals involved in this process [4]. It is essential to emphasize to the parents-to-be that there is nothing they have done to account for the cleft, because feelings of guilt are very harsh [2]. However, when the diagnosis is known before delivery, the parents would have a chance to go through the grieving process [2].

Health professionals are expected to have accurate and clear answers on possible questions posed by the parents following ultrasonographic diagnosis [4, 5]. The responsibility of the referral center is to define the nature of the structural defect with as much precision as possible. Furthermore, giving the information of burden of treatment and prognosis regarding a possible less-than-ideal outcome depending on the severity is critical to the process of counseling. Recurrence risk may also be one of the major concerns the parents and families may have to confront [4]. Families who have a previous child with CL ± CP have a 3 to 5 percent chance of recurrence, and if a parent and a previous child are both affected, the recurrence rate may be as high as 15 percent [3].

### Summary

Prenatal diagnosis of orofacial cleft gives the parents the possibility to prepare themselves in an emotional and practical way [20]. A frank discussion of the cost of care and strategies for obtaining coverage for care can also relieve unspoken anxiety [4]. Knowing the diagnosis before delivery also allows the cleft team to discuss the plans, for example, feeding issues, type and geographical location of the cleft [2]. This would make delivery much less traumatic, especially for the parents and the extended family [2]. Our department offers information pamphlet for the future parents when they visit for first counseling. Information pamphlet should contain the concerns of parents-to-be, including longitudinal management of clefts and contact numbers [2].

For an effective counseling, accuracy of diagnosis is essential. Diagnosing techniques have been dramatically improved throughout the decades by technology, equipment and skills. However, health professionals must be aware of the possible pitfalls and related considerations of the ultrasonographic diagnosis for better treatment planning and counseling.

## Consent

Written informed consent was obtained from the parents of the patients for publication of this Case report and any accompanying images. A copy of the written consent is available for review by the Editor-in-Chief of this journal.

## Abbreviations

CL: Cleft lip
CP: Cleft palate
CL + CP: Cleft lip with cleft palate
CL ± CP: Cleft lip with or without cleft palate
